# Functionalities of Gelatin Modified with 2-Octenyl Succinic Anhydride and Gallic Acid

**DOI:** 10.3390/foods11091241

**Published:** 2022-04-26

**Authors:** Tai-Ti Liu, Xin-Yi Zhuang, Tsung-Shi Yang

**Affiliations:** 1 Department of Food Science, Yuanpei University of Medical Technology, No. 306 Yuanpei Street, Hsinchu 30015, Taiwan; em46483218@gmail.com; 2 Department of Cosmeceutics, China Medical University, No. 100, Sec. 1, Jingmao Rd., Beitun Dist., Taichung 406040, Taiwan

**Keywords:** gelatin, 2-octenyl succinic anhydride, surface activity, gallic acid, antioxidant activity, antimicrobial activity

## Abstract

The aim of this research was to modify gelatin (GT) with 2-octenyl succinic anhydride (OA) and gallic acid (GA) and investigate its functionalities. GT modified with OA (GT-OA) has an improved water solubility at room temperature and an enhanced surface activity, foaming capacity, and pH buffering ability. Regarding antioxidant activity, GT-OA grafted with GA to generate the compound GT-OA-GA has shown good antioxidant activity. Particularly, GT-OA-GA surpassed GA in ferrous ion (Fe^2+^)-chelating activity. With respect to antimicrobial activity, GT-OA-GA could be complexed with zinc ions (Zn^2+^), and this complex exhibited good antimicrobial activity against *Staphylococcus aureus* and *Escherichia coli* (O157:H7). Chemically modified GT has better water solubility at room temperature and more functionalities than unmodified GT. Thus, it can be used as an emulsifier or coating material in food, cosmetic, and pharmaceutical industries pertaining to GT applications.

## 1. Introduction

Gelatin (GT) is a protein produced from collagen-containing tissues via an acid (type A) or alkaline (type B) pretreatment and a subsequent extraction. It is one of the most versatile biopolymers and it is widely used in food, confectionery, pharmaceutical, medical, cosmetic, and technical products [1]. Because it contains more hydrophilic amino acids than hydrophobic residues, GT is essentially hydrophilic, which limits its surface activity [2]. In addition, the poor water solubility of GT at room temperature often causes problems for its application in many food and pharmaceutical manufacturing processes [3].

Modifying the chemical structure of GT can improve its defects or enhance its functionalities. Regarding the hydrophobicity and surface activity enhancement of GT, Shilpi et al. [4] conjugated GT to stearic acid activated by 1-ethyl-3-(3-dimethylaminopropyl) carbodiimide (EDC)/N-hydroxysuccinimide (NHS) to increase the loading efficiency of a drug with poor aqueous solubility. Using a similar activation method, Nguyen et al. [5] synthesized a GT-oleic acid conjugate, which could form self-assembled nanoparticles. The conjugate has a hydrophobic inner core for loading water-insoluble drugs and a GT layer for biocompatibility and circulation extension. The role of hydrophobic molecules in an amphiphilic polymer is important for preparing nanoparticles because it may affect the particle size, drug loading efficiency, and stability.

Succinylation is another frequently used method for protein modification. Commercial bovine GT can be modified by using succinic anhydride, which changes the physicochemical properties of GT, such as foaming and bloom strength [6]. Octenyl succinic anhydride (OA) has been used to modify bovine bone GT (type B) and fish skin GT, thus increasing the droplet stability, phase transition time, and creaming index of fish oil-loaded emulsions [7]. Porcine skin GT (type A) conjugated to 2-octenyl succinic anhydride; however, this has not been studied.

Protein-conjugated polyphenols have been widely reported [8]. Regarding the conjugation of GT with polyphenols, GT has been modified with epigallocatechin gallate for medical applications [9]. In addition, polyphenols from plant extracts were used to cross-link GT molecules to enhance their gel strength [10]. A GT-gallic acid conjugate has been fabricated to evaluate its applicability in biomedicine and pharmacy [11]. Cuttlefish skin GT modified with caffeic acid, ferulic acid, or tannic acid was also investigated. In the study, the antioxidant activity of the modified GT increased, but the surface hydrophobicity decreased [12].

The aim of this research was to conjugate GT (type A) with 2-octenyl succinic anhydride and GA to improve the solubility of GT at room temperature and increase its functionalities, such as emulsifying, antioxidant, and antimicrobial activities.

## 2. Materials and Methods

### 2.1. Materials

GT from porcine skin (type A, bloom# 300), OA, GA, 2,2-diphenyl-1-picrylhydrazyl (DPPH), Folin–Ciocalteu’s phenol reagent, zinc nitrate hexahydrate, and ninhydrin were obtained from Sigma-Aldrich Co. (St. Louis, MO, USA). Ferrozine, EDC, and NHS were purchased from Alfa Aesar Co. (Ward Hill, MA, USA). Ferric chloride hexahydrate was acquired from Merck (Darmstadt, Germany). Potassium ferricyanide and trichloroacetic acid were purchased from Ferak Berlin GmbH (Berlin, Germany). Ferrous chloride tetrahydrate was obtained from J. T. Baker (Center Valley, PA, USA). Tryptic soy broth, nutrient broth, and agar were acquired from Difco Laboratories (Detroit, FL, USA).

### 2.2. Microbial Strains

*Staphylococcus aureus* (BCRC 10781) and *Escherichia coli* (O157: H7; BCRC 14824) were obtained from the Bioresource Collection and Research Center (BCRC) at the Food Industry Research and Development Institute in Hsinchu, Taiwan.

### 2.3. Synthesis of GT and OA Conjugate (GT-OA)

GT (1 g) was added into 22 mL sodium hydroxide solution (0.01 N). Double deionized water was used in all experiments unless specified. The solution was heated at 70 °C for 30 min with stirring for complete dissolution. Next, OA was slowly added with stirring within 1 h of mixing. The pH of the solution was adjusted to 8, and the solution was allowed to react for 5 h [13]. After reaction, the solution was cooled to room temperature and dialyzed via a tube membrane (12–14 kD MWCO) immersed in 25% (*v/v*) aqueous alcohol solution for 1 d, and then in water for 2 d while stored at 4 °C with periodic water replacement using clean water to remove the unreacted reagents and by-products. Subsequently, this solution was freeze-dried to obtain the synthesized product.

### 2.4. Synthesis of GT-OA-GA

**Figure 1:**
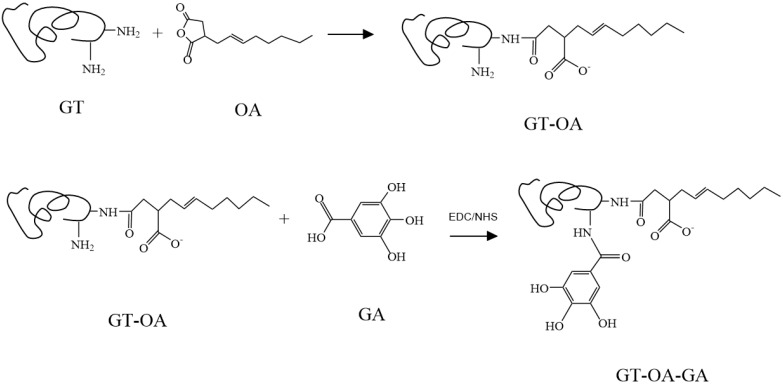
Syntheses of GT-OA and GT-OA-GA.

GT modified with OA (GT-OA) (0.25 g) was added into 20 mL water and the pH was adjusted to 3. GA and EDC/NHS were dissolved in 50 mL methanol at the designed ratios and stirred for 1 h. This solution was then slowly added to the GT-OA solution with stirring and allowed to react in the dark for 24 h in an ice-water bath. Afterward, the resultant mixture was dialyzed in water, as described above, and lyophilized to acquire the synthesized product. The structure of GT conjugated to OA and GA (GT-OA-GA) is depicted in Figure 1.

### 2.5. NMR Analysis

GT, GT-OA, and GT-OA-GA were dissolved in deuterium oxide (D_2_O), and OA was dissolved in deuterated chloroform (CDCl3). The proton nuclear magnetic resonance (^1^H-NMR) spectra of these samples were analyzed by an NMR spectrometer (Agilent DD2, 600 MHz).

### 2.6. Degree of Amino Group Substitution of GT

The degree of amino group substitution of GT was determined according to the modified method of Shilpashree et al. [14]. The sample (0.02 g) was dissolved in a 0.1 N NaOH solution, of which 100 µL was added to a microtube. Afterwards, 100 µL water was added into the tube, followed by the addition of 200 µL ninhydrin (0.5%, *v/v*). The microtube was then heated at 100 °C for 15 min and cooled in an ice-water bath for 5 min. Next, 600 µL ethanol (50%, *v/v*) was added into the tube, which was shaken for 5 s and then centrifuged at 2500× *g* for 15 min. The clear solution was measured at an absorbance of 570 nm with a microplate reader (M200PRO, Tecan, Männedorf, Switzerland). The degree of amino group substitution of GT was calculated using the following Equation (1):

The degree of amino group substitution of GT (%) = ((A − B)/A) x 100(1)

where A and B are the absorbance of unmodified and modified GT, respectively.

### 2.7. Surface Tension Measurement

A 10 mL sample solution (2 mg/mL) was added to a round glass dish (ID: 5 cm) on a movable stage. Surface tension was measured based on the Du Noüy ring method. A platinum ring attached to a tensiometer (KSV Sigma 703D, Biolin Scientific, Stockholm, Sweden) was then immersed in the liquid. Slowly adjusting the stage downward caused the ring to raise the meniscus of the liquid. When the meniscus tore from the ring, the maximal pulling force was recorded. Accordingly, the surface tension was automatically calculated by the tensiometer [15].

### 2.8. Foaming Properties

An aqueous sample solution (1%, *w/v*) was subjected to foaming with a homogenizer at 6000 rpm for 6 min. Then, the sample was quickly transferred to a 25 mL graduated cylinder and stored in an incubator at 30 °C for 30 min [14]. Foam capacity and stability were determined by Equations (2) and (3), respectively:

Foam capacity (%) = ((VT − Vo)/Vo) × 100(2)

where Vo is the volume of liquid before whipping (mL) and VT is the total volume (foam plus liquid) obtained immediately after whipping (mL)

Foam stability (%) = ((Vs)/VT) × 100(3)

where VT and Vs are foam volumes before and after storage, respectively.

### 2.9. pH Buffering Capacity

The 10 mL sample solution (1%, *w/v*) was placed in a beaker with stirring, and then titrated with 0.01 N hydrochloric acid (HCl). The volume of the added titrant and pH were recorded, and their relation was plotted to indicate the pH buffering capacity of the sample.

### 2.10. Antioxidant Activity

#### 2.10.1. DPPH Radical Scavenging Activity

The sample (50 μL) was mixed with 150 μL DPPH in methanol (0.25 mM) in a 96-well microplate and left undisturbed for 10 min at room temperature. Then, the absorbance was measured at 517 nm [16]. The DPPH scavenging activity was calculated using Equation (4) as follows:

Scavenging activity (%) = ((Ac − As)/Ac) × 100(4)

where Ac is the absorbance of the control and As is the absorbance of the sample.

#### 2.10.2. Chelating Activity of Ferrous Ions

The sample (200 µL) was thoroughly mixed with 200 µL ferrous chloride (FeCl_2_) (0.18 mM) at 37 °C for 1 h in the dark, followed by the addition of 100 μL ferrozine (0.72 mM), and reacted for 5 min. This solution was then centrifuged at 8000× *g* for 10 min, and the clear solution was measured at an absorbance of 562 nm [17]. The chelating activity of the ferrous ions was determined using the Equation (5) as follows:

Chelating activity (%) = ((Ac − As)/Ac) × 100(5)

where Ac is the absorbance of the control and As is the absorbance of the sample.

#### 2.10.3. Ferric Ion Reducing Power

The sample (100 μL) was mixed with 100 μL phosphate buffered saline (PBS) (0.2 M, pH 6.6) and 100 μL potassium ferricyanide (1%, *w/v*), and then incubated at 50 °C for 20 min. Afterward, 100 μL trichloroacetic acid (10%, *w/v*) was added to the mixture, which was then centrifuged at 4000× *g* for 5 min. The solution (100 μL) was then added to 100 μL of distilled water and 30 μL of ferric chloride (0.1%, *w/v*) and reacted for 10 min. The absorbance of the reaction mixture was measured at 700 nm [18]. The control contained all reagents except the sample, which was replaced by water. The larger the absorbance, the higher the reducing power became.

### 2.11. Complexation of GT Conjugates with the Zinc Ion

GT conjugates (0.1 g) were dissolved in 2 mL water, and 1 mL zinc nitrate (Zn[NO_3_]_2_) was added at a series of concentrations. The pH of the solution was adjusted to 7, and the solution was stored in the dark for 3 d. Then, the solution was lyophilized to obtain the complexed product.

### 2.12. Antimicrobial Activity Assay

The bacteria from stock cultures were inoculated in 5 mL broth and incubated at 37 °C for 24 h to increase the microbial population. The concentration of the enriched cultures was controlled with dilution to an optical density of 1 at 600 nm. Cultures were further diluted with the necessary medium for each microbe to ca. 1 × 10^2^ colony forming units (CFU/mL) as an inoculum. The media for *S. aureus* and *E. coli* O157:H7 were tryptic soy broth (3%, *w/v*) and nutrient broth (0.8%, *w/v*), respectively.

A microbial suspension was mixed with the test sample (1:1, *v/v*) at a series of concentrations in a 1.5 mL sterilized microtube. The tubes were tightly capped and incubated for 48 h at 37 °C. The minimum bactericidal concentration (MBC) was determined by visually judging the microbial growth in the tubes with a series of broth dilutions. Then, the existence of viable cells was verified by a plate count method. The MBC is defined as the lowest concentration of the sample that results in no viable cells in a culture plate [19]. An inoculated growth medium without the tested compound was employed as a control. All the experiments were performed in triplicate.

### 2.13. Statistical Analysis

Data were statistically analyzed by analysis of variance (ANOVA) using Statistica for Windows (StatSoft, Tulsa, OK, USA). The mean data values were compared based on Duncan’s multiple range test with significant differences set at *p* < 0.05.

## 3. Results and Discussion

### 3.1. Synthesis of GT-OA and GT-OA-GA

GT was conjugated to OA to form GT-OA via octenyl succinylation, by which OA reacts mainly with the ε-amino group of lysine and slightly with the N-terminal amino acids in the protein. Thus, the positively charged residues of the protein were changed to negatively charged residues via N-acylation [7]. The amino group substitution (%) was used to evaluate the efficiency of the conjugation because the amino groups of GT are the major reactive sites. The effects of the reactants at different ratios for the synthesis of GT-OA are listed in Table 1. The amount of GT remained constant in the experiment, while the OA concentration was varied (Table 1). To determine if the reaction process would affect the properties of GT, we allowed the GT to go through the same process in the absence of OA. Then, the amino group substitution was determined based on the treated GT rather than the original GT. When the ratio of OA to GT increased, the amino group substitution also increased. This meant that more OA was grafted onto GT. The amino group substitution increased by approximately 5–7% for each additional amount of OA that was used. Although the amino group substitution of GT-OA increased, the product yield decreased. For example, when the amino group substitution increased from 70.88% (GT-OA-1) to 80.10% (GT-OA-3), the yield was reduced from 65.02% (GT-OA-1) to 40.89% (GT-OA-3). Comparatively, the increase in the amino group substitution was much less than the yield loss of the synthesized product. The reason is likely that the reactive sites of the GT gradually decreased during the reaction period, which reduced the collision probability between the sites and reactants. Moreover, the grafted OA might interact with the other OA molecules via hydrophobic attraction to deter their approach to the residual reactive GT sites. Thus, the unreacted OA molecules were hydrolyzed in the aqueous solution [20] and removed in the later dialytic process. Considering the amino group substitution and yield, GT-OA-2 was used for conjugation with GA.

### 3.2. NMR Spectra of GT-OA and GT-OA-GA

**Figure 2:**
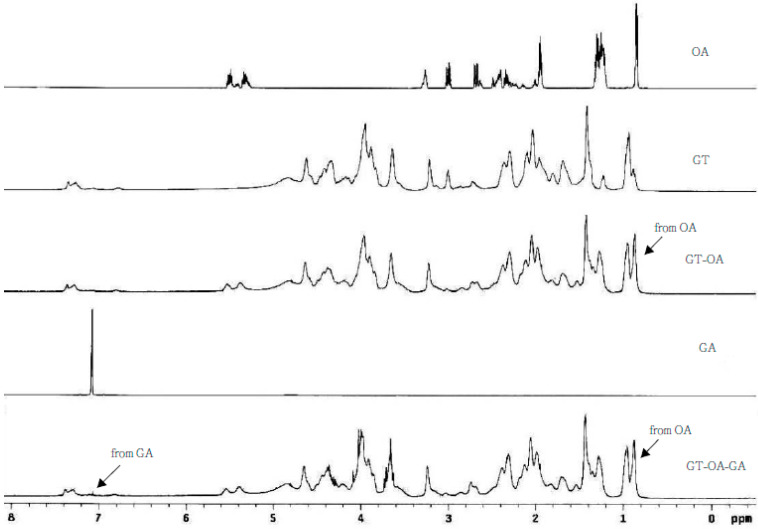
NMR spectra of OA, GT, GT-OA, GA, and GT-OA-GA.

The NMR spectra of OA, GT, GT-OA, GA, and GT-OA-GA are displayed in Figure 2. Compared with the GT spectrum, the signal of the peak in the chemical shift located at 0.9 ppm in the GT-OA spectrum was greatly enhanced, which resulted from the methyl group of OA. This indicates the successful grafting of OA onto GT. Moreover, a new peak was shown in the chemical shift located at 7.2 ppm in the GT-OA-GA spectrum, which corresponded to that of GA (phenol group), as compared with the spectra of GT and GT-OA. This also demonstrates that GA was chemically bonded onto GT-OA.

### 3.3. Characterization of GT-OA

#### 3.3.1. Surface Activity, Zeta Potential, and Viscosity

The presence of OA in the GT structure influenced the physicochemical properties of GT. Regarding surface activity, the incorporation of OA into GT could increase the surface activity of GT. The more OA that was used, the higher the surface activity of GT-OA became, as shown from the surface tension reduction in Table 1. However, the degree of surface tension reduction was not proportional to the amount of OA added. Although the carbon chain of OA could increase the hydrophobicity of GT-OA, the carboxylate group of OA also provided some hydrophilicity, which may have somewhat offset the lipophilic effect of OA.

When OA is conjugated onto GT, one carboxyl group of OA reacts with the amino group of GT to form a covalent bond, while the other carboxylate group remains free, resulting in a negative charge in the aqueous solution (Figure 1). Before succinylation, the zeta potential of GT was positive, but after the addition of OA in the reaction, the zeta potential became negative. The zeta potential value increased with the increase of OA in the reaction. Moreover, the zeta potential might affect the viscosity of GT-OA. For example, GT is a gel forming molecule; thus, the greater the interactive forces among the protein molecules, the higher the viscosity of the solution. The results in Table 1 show that GT had the highest viscosity, and the viscosity of GT-OA decreased as the amount of OA increased. The reason for this may be ascribed to the increase in the zeta potential, which represents an increase in the negative charge. The negative charges among the GT-OA molecules generate repulsive forces that might weaken the protein-protein interactions and thereby reduce the viscosity [21].

#### 3.3.2. Foaming Properties

The results showed that the foaming capacity of GT-OA was better than that of GT (Figure 2). This indicated that OA could enhance the foaming capacity of GT after conjugation. High hydrophobicity and viscosity have been reported to favor protein foaming capacity [22]. The carbon chain of OA provided the hydrophobicity for GT-OA. Thus, the more OA that was conjugated onto GT, such as in GT-OA-1 and GT-OA-2, the higher the foaming capacity of the GT-OA conjugate became. However, the foaming capacity seemed to level off when OA was further increased in the conjugates, such as in GT-OA-3, GT-OA-4, and GT-OA-5. Viscosity did not play an important role in this experiment because the foaming capacity did not decrease despite the decreased viscosity of GT-OA compared with GT. It is possible that the differences in viscosity between the GT and GT-OA conjugates in this experiment were not large enough to cause a significant effect as compared to the increased hydrophobicity effect of OA on the foaming capacity. More importantly, the surface activity may be a more critical factor in determining the foaming capacity. GT-OA conjugates have a greater surface activity than that of GT. The reduction of the surface tension between the air and water phases facilitates foam formation by removing the energy barrier. Lawal [23] reported that a progressive increase in the level of succinylation of *Lablab* bean protein concentrates with succinic anhydride increased the foam capacity. The authors suggested that acylation could cause protein unfolding and increase protein-water interactions. In addition, the increased net negative charge of succinylated proteins could promote protein-water interactions and thus improve foaming capacity.

**Figure 3:**
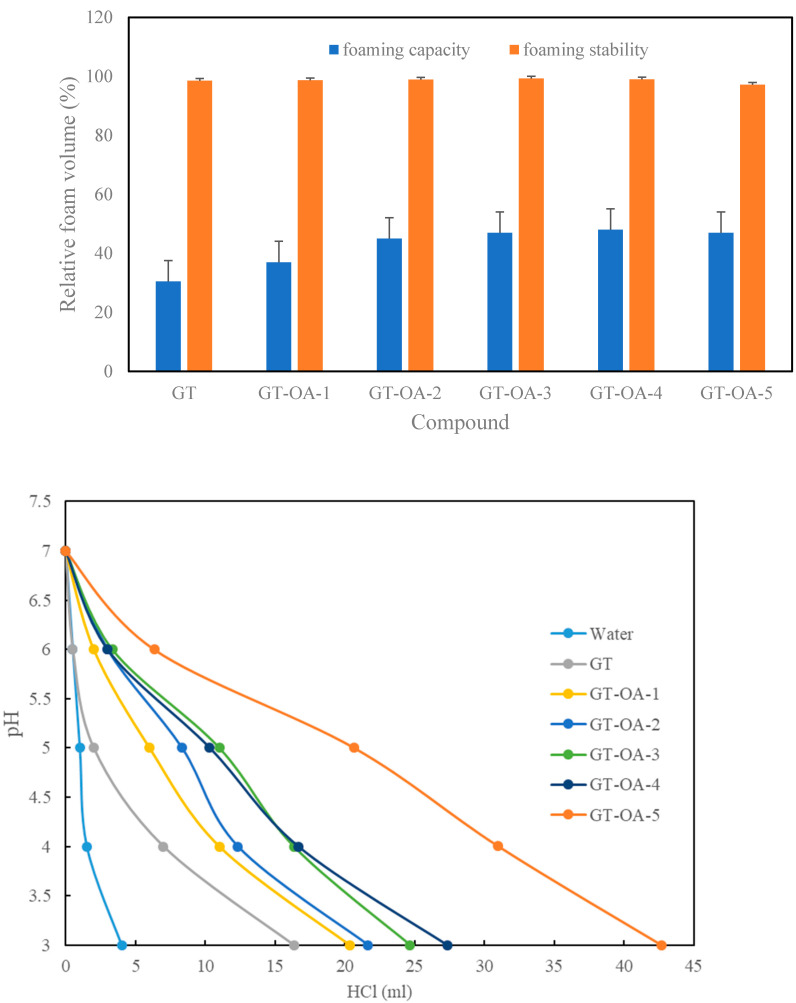
The foaming capacity and stability (**above**), and pH buffering capacity (**bottom**) of GT and GT-OA synthesized in different conditions.

Regarding foaming stability, flocculation is a precursor to the destabilization of bubbles. Flocculation brings about the deformation of colliding bubbles. The deformed bubbles are then separately surrounded by a thin liquid film. The intersection of three such films can form a channel called a plateau border. Liquid drains from the thin film to the plateau border causing bubble instability. One of the driving forces for liquid drainage is called plateau border suction, which is equal to the ratio of the surface tension to the curvature radius of the plateau border. Therefore, reducing the surface tension can decrease the drainage force. Emulsifiers and proteins stabilize foams by reducing the surface tension, thereby lowering the rate of thin film drainage [24]. The foaming stability of GT and all GT-OA conjugates appeared very stable during the experimental period (Figure 3). The higher surface-tension-reducing ability might be a booster for the foaming stability of the GT-OA conjugates. In contrast, the higher viscosity might be an important contributor for the foaming stability of GT.

#### 3.3.3. pH Buffering Capacity

The pH buffering capacity is the ability of a compound to resist pH changes. Because GT-OA carries more carboxylic groups derived from the structure of OA, they are negatively charged at neutral or alkaline conditions. Consequently, protonation of the GT-OA conjugates was performed by titrating the solution of the conjugates with HCl. The amounts of HCl used were recorded and plotted with the pH (Figure 3). The more titrant that was used to reach the same pH, the higher the buffering capacity was of the compound. As shown in Figure 3, the more OA that was conjugated to GT, the larger the volume of HCl that was required to reach the same pH value. OA provides the carboxyl group that can be protonated; thus, GT grafted with more OA exhibited a greater pH buffering capacity.

### 3.4. Antioxidant Activity of GT Conjugates

#### 3.4.1. DPPH Radical Scavenging Activity

The DPPH assay is frequently used in the evaluation of radical scavengers due to its simple and rapid manual analysis. DPPH is a stable free radical with a purple color, and it can be reduced upon acceptance of a hydrogen atom or electron from an antioxidant, which causes it to become decolorized to a yellow compound [25]. GT or GT-OA did not show any scavenging effect on DPPH, but GT-OA with GA conjugates all showed antioxidant activity. This indicates that the antioxidant activity of GT-OA-GA conjugates mainly came from GA because GA alone exhibited excellent antioxidant activity. In addition, GT-OA-GA-Zn had a lower DPPH scavenging activity than GT-OA-GA. The reason for this might be that a part of the GA OH groups was used to form a complex with zinc ions (Zn^2+^), which affected its electron-transferring activity.

#### 3.4.2. Ferrous Ion Chelating Activity

The ferrous ion (Fe^2+^) is an important initiator for oxidation. Namely, Fe^2+^ can produce a hydroxyl radical, which is the most reactive species of oxygen, via the Fenton reaction. The half-life of the hydroxyl radical in biological systems is about 1 ns, and it can rapidly react with organic molecules with rate constants of 10^9^–10^10^ M^−1^ s ^−1^ [26]. Therefore, the Fe^2+^-chelating activity of the GT conjugates was tested. As shown in Table 2, GT, GA, and GT-OA did not display Fe^2+^-chelating activity in the tested concentration range. However, when GA was grafted onto GT-OA, GT-OA-GA showed marked chelating activity as compared with the individual compounds. The OH group of GA may play an important role in this chelating effect. Similarly, GA did not show Fe^2+^-chelating activity in previous reports [16,27]. However, when GA was conjugated to chitosan, the chelating activity of this conjugated compound was markedly elevated [16]. Chelation generally requires unpaired electrons to attract positive metal ions, and some atoms, such as oxygen and nitrogen, can provide these electrons. Therefore, the location and orientation of these atoms in the molecular structure might determine the chelating effect. In this case, even though OA has a free carboxyl group, its location and orientation might not favor this effect on GT-OA. Nevertheless, favorable chelating conditions might be created with the involvement of the OH groups of GA in the nearby structure after conjugation. When GT-OA-GA was complexed with Zn^2+^, its chelating activity was slightly influenced (Table 2). It is reasonable to infer that part of the chelating site of GT-OA-GA was occupied by Zn^2+^ ions, which resulted in the decrease.

#### 3.4.3. Ferric Ion Reducing Antioxidant Power Assay

Because standard one-electron reduction potentials can be used to predict the ability of a free radical scavenger to donate hydrogen to a free radical [28], the electron reducing ability of GT conjugates was also investigated. From Table 2, GT and GT-OA did not show any electron reducing effect, but GA and GT-OA-GA did. This also demonstrates that GA is the key compound responsible for this type of antioxidant activity. The ferric ion reducing ability, expressed as an ascorbic acid or GA equivalent, was not significantly different between GT-OA-GA and GT-OA-GA-Zn, which differed slightly from the result of the DPPH assay. The reason for this might be that the sample amounts used for the ferric ion reducing assay were much higher (approximately twice as much) than those used for the DPPH assay. Thus, the influence of the increasing effect of GA might be higher than that of Zn^2+^ because GA displayed a stronger reducing ability than ascorbic acid, which is a good reducing compound (Table 2).

### 3.5. Complexation of GT Conjugates with the Zinc Ion and Antimicrobial Activity of the Complexes

**Figure 4:**
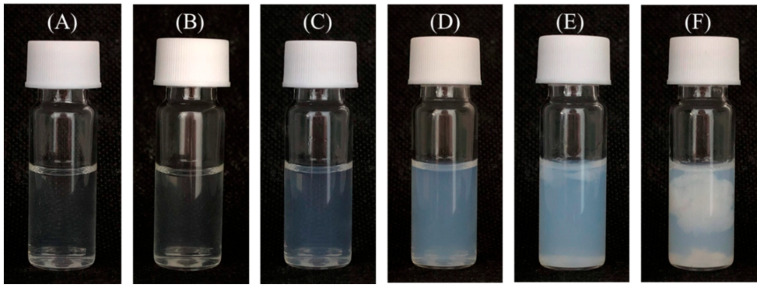
Appearances of GT-OA mixed with Zn(NO_3_)_2_ solution at a series of concentrations (mM): (**A**) 16.8, (**B**) 20.2, (**C**) 23.5, (**D**) 26.9, (**E**) 30.3, (**F**) 33.6.

GT-OA was added to a series of Zn(NO_3_)_2_ concentrations (16.8–33.6 mM) to form a complex. When the Zn(NO_3_)_2_ concentration reached 23.5 mM, the solution became slightly turbid with a colloidal suspension. The turbidity of the solution gradually increased as the Zn(NO_3_)_2_ concentration increased. The solution formed heavy precipitates at the highest concentration (Figure 4). This indicates that GT-OA and Zn^2+^ interact mainly via electric attraction. Therefore, the 20.2 mM Zn(NO_3_)_2_ concentration, which was the highest concentration remaining in a clear solution, was used for the complexation of the GT conjugates for subsequent antimicrobial activity assays in this experiment.

The results in Table 3 show that the GT conjugates without Zn^2+^ did not have antimicrobial activity. This means that Zn^2+^ is the key component that confers antimicrobial activity to the GT conjugates. The amount of Zn(NO_3_)_2_ in the complex of the GT conjugates was estimated to be approximately 3.68%, assuming no significant loss occurred during the experimental process. As a result, the MBC of GT-OA-Zn for *S. aureus* was 5000 µg/mL and contained Zn(NO_3_)_2_ at a concentration of about 184 µg/mL, which was higher than the MBC of Zn(NO_3_)_2_ (75 µg/mL). The reason for this is probably that a large portion of the Zn^2+^ ions are temporally adsorbed in the molecular structure of the GT conjugates so that they are not released during the change in chemical equilibrium. However, the Zn^2+^ ions that are retained in the GT conjugates might undergo a controlled release by the dynamic change of chemical equilibrium in different conditions.

## 4. Conclusions

GT conjugated to OA can improve its water solubility at room temperature and enhance its surface activity. The presence of OA in the GT structure affects the zeta potential, viscosity, foaming property, and buffering capacity of GT-OA. With additional grafting of GA onto GT-OA, this conjugate can be imparted with antioxidant abilities. In particular, the Fe^2+^ chelating ability, which pure GA lacks, is generated via this conjugation. GT-OA-GA can be further complexed with Zn^2+^ and rendered antimicrobial activity. This study provides useful information about optional ways of modifying GT to fit various purposes of application. For example, the modified GT with surface activity and antioxidant activity can be used in oil-in-water emulsions as an emulsifier to stabilize oxidatively labile compounds or as a coating material to encapsulate functional ingredients in food, cosmetic, or pharmaceutical industries pertaining to GT applications.

## Figures and Tables

**Table 1:** Effects of reactant ratios on grafting efficiency of the GT-OA synthesis and the physicochemical properties of GT-OA.

Sample	GT (g)	OA (g)	Amino-Substitution (%)	Surface Tension Reduction (%) ^1^	Zeta Potential (mv)	Viscosity (cp)	Yield (%)
GT	1	0	-	22.81 ± 0.31 ^e,2^	0.28 ± 0.72 ^a^	4.67 ± 0.11 ^a^	77.82 ± 0.61 ^a^
GT-OA-1	1	0.5	70.88 ± 0.18 ^e^	24.53 ± 0.65 ^d^	−13.63 ± 0.21 ^b^	3.98 ± 0.05 ^b^	65.02 ± 0.74 ^b^
GT-OA-2	1	1	76.41 ± 0.44 ^d^	25.05 ± 1.63 ^d^	−14.43 ± 0.25 ^c^	3.66 ± 0.23 ^c^	50.29 ± 1.01 ^c^
GT-OA-3	1	1.5	80.10 ± 0.42 ^c^	28.46 ± 0.79 ^c^	−17.47 ± 0.15 ^d^	3.12 ± 0.24 ^d^	40.89 ± 0.53 ^d^
GT-OA-4	1	2	84.29 ± 0.43 ^b^	30.18 ± 0.76 ^b^	−19.03 ± 0.12^e^	2.94 ± 0.18 ^de^	32.90 ± 0.72 ^e^
GT-OA-5	1	4	90.02 ± 0.68 ^a^	32.29 ± 0.93 ^a^	−20.93 ± 0.32 ^f^	2.70 ± 0.05 ^e^	20.16 ± 0.63 ^f^

^1^: compared with the surface tension of water; ^2^: the values in the same column with different letters are significantly different at *p* < 0.05.

**Table 2:** Reactant ratios for the synthesis of GT-OA-GA and antioxidant activities of GT-OA-GA as well as its complex determined by different assays.

Sample	GT-OA (g)	GA (mmol)	EDC (mmol)	NHS (mmol)	Assays (IC50)			
					DPPH (mg/mL)	Fe^2+^-Chelating Power (μg/mL)	Fe^3+^-Reducing Power (Ascorbic Acid, μg/mL) ^1^	Fe^3+^-Reducing Power (Gallic Acid, μg/mL) ^1^
GT	-	-	-	-	-	>25,000	-	-
GA	-	-	-	-	0.0062 ± 0.0001	>25,000	-	-
GT-OA	-	-	-	-	>10	>25,000	-	-
GT-OA-GA	0.25	2.4	2.4	2.4	1.79 ± 0.02 ^b,2^	225.41 ± 5.33 ^b^	96.71 ± 2.71 ^a^	52.32 ± 1.46 ^a^
GT-OA-GA-Zn	0.25	2.4	2.4	2.4	2.34 ± 0.06 ^a^	241.23 ± 7.32 ^a^	92.43 ± 8.37 ^a^	50.02 ± 4.52 ^a^

^1^: ascorbic acid or gallic acid equivalent of GT-OA-GA and GT-OA-GA-Zn at 5 mg/mL; ^2^: the values in the same column with different letters are significantly different at *p* < 0.05.

**Table 3:** Minimal bactericidal concentrations of the Zn^2+^ complexes of GT conjugates against *S. aureus* and *E. coli.*

Bacterium	Minimal Bactericidal Concentration (µg/mL)					
	GT	GT-OA	GT-OA-GA	GT-OA-Zn	GT-OA-GA-Zn	Zn (NO_3_)_2_
*S. aureus*	-	-	-	5000	5000	75
*E. coli*	-	-	-	1250	2500	18.75

## Data Availability

Data is contained within the article.

## References

[1] Haug I.J., DragetPhillips K.I., Phillips G.O., Williams P.A. (2011). Gelatin. Handbooks of Food Proteins.

[2] Toledano O., Magdassi S. (1997). Formation of surface active gelatin by covalent attachment of hydrophobic chains. J. Colloid Interface Sci..

[3] Ghorani B., Emadzadeh B., Rezaeinia H., Russell S.J. (2020). Improvements in gelatin cold water solubility after electrospinning and associated physicochemical, functional and rheological properties. Food Hydrocoll..

[4] Shilpi D., Kushwah V., Agrawal A.K., Jain S. (2017). Improved stability and enhanced oral bioavailability of atorvastatin loaded stearic acid modified gelatin nanoparticles. Pharm. Res..

[5] Nguyen V.H., Lee B.J. (2017). Synthetic optimization of gelatin-oleic conjugate and aqueous-based formation of self-assembled nanoparticles without cross-linkers. Macromol. Res..

[6] Alias N.A.B., Omosebi B.O., David W., Huda N. (2017). Improving the Physicochemical Properties of Commercial Bovine Gelatin using Succinylation. Asia Pac. J. Sustain. Agric. Food Energy.

[7] Zhang T., Xu J., Zhang Y., Wang X., Lorenzo J.M., Zhong J. (2020). Gelatins as emulsifiers for oil-in-water emulsions: Extraction, chemical composition, molecular structure, and molecular modification. Trends Food Sci. Technol..

[8] Quan T.H., Benjakul S., Sae-leaw T., Balange A.K., Maqsood S. (2019). Protein–polyphenol conjugates: Antioxidant property, functionalities and their applications. Trends Food Sci. Technol..

[9] Honda Y., Takeda Y., Li P., Huang A., Sasayama S., Hara E., Uemura N., Ueda M., Hashimoto M., Arita K. (2018). Epigallocatechin gallate-modified gelatin sponges treated by vacuum heating as a novel scaffold for bone tissue engineering. Molecules.

[10] Zhao Y., Sun Z. (2017). Effects of gelatin-polyphenol and gelatin–genipin cross-linking on the structure of gelatin hydrogels. Int. J. Food Prop..

[11] Cirillo G., Kraemer K., Fuessel S., Puoci F., Curcio M., Spizzirri U.G., Altimari I., Iemma F. (2010). Biological activity of a gallic acid− gelatin conjugate. Biomacromolecules.

[12] Aewsiri T., Benjakul S., Visessanguan W., Eun J.-B., Wierenga P.A., Gruppen H. (2009). Antioxidative activity and emulsifying properties of cuttlefish skin gelatin modified by oxidised phenolic compounds. Food Chem..

[13] Song X., Zhao Q., Li Z., Fu D., Dong Z. (2013). Effects of amylose content on the paste properties and emulsification of octenyl succinic starch esters. Starch-Stärke.

[14] Shilpashree B., Arora S., Chawla P., Tomar S. (2015). Effect of succinylation on physicochemical and functional properties of milk protein concentrate. Food Res. Int..

[15] Liu T.T., Su G.Z., Yang T.S. (2021). Functionalities of chitosan conjugated with lauric acid and l-carnitine and application of the modified chitosan in an oil-in-water emulsion. Food Chem..

[16] Yang T.S., Liu T.T., Lin I.H. (2017). Functionalities of chitosan conjugated with stearic acid and gallic acid and application of the modified chitosan in stabilizing labile aroma compounds in an oil-in-water emulsion. Food Chem..

[17] Santos J.S., Brizola V.R.A., Granato D. (2017). High-throughput assay comparison and standardization for metal chelating capacity screening: A proposal and application. Food Chem..

[18] Girgih A.T., He R., Hasan F.M., Udenigwe C.C., Gill T.A., Aluko R.E. (2015). Evaluation of the in vitro antioxidant properties of a cod (*Gadus morhua*) protein hydrolysate and peptide fractions. Food Chem..

[19] Yang T.-S., Liou M.-L., Hu T.-F., Peng C.-W., Liu T.-T. (2013). Antimicrobial activity of the essential oil of Litsea cubeba on cariogenic bacteria. J. Essent. Oil Res..

[20] Hui R., Qi-He C., Ming-liang F., Qiong X., Guo-qing H. (2009). Preparation and properties of octenyl succinic anhydride modified potato starch. Food Chem..

[21] Adebowale K.O., Lawal O.S. (2003). Foaming, gelation and electrophoretic characteristics of mucuna bean (*Mucuna pruriens*) protein concentrates. Food Chem..

[22] Townsend A.A., Nakai S. (1983). Relationships between hydrophobicity and foaming characteristics of food proteins. J. Food Sci..

[23] Lawal O.S. (2005). Functionality of native and succinylated Lablab bean (*Lablab purpureus*) protein concentrate. Food Hydrocoll..

[24] Narsimhan G., Xiang N. (2018). Role of proteins on formation, drainage, and stability of liquid food foams. Annu. Rev. Food Sci. Technol..

[25] Dontha S., Kamurthy H., Manthriparagada B. (2016). Phytochemical screening and evaluation of in-vitro antioxidant activity of extracts of Ixora javanica DC flowers. Am. Chem. Sci. J..

[26] Buxton G.V., Greenstock C.L., Helman W.P., Ross A.B. (1988). Critical Review of rate constants for reactions of hydrated electrons, hydrogen atoms and hydroxyl radicals (⋅ OH/⋅ O− in Aqueous Solution. J. Phys. Chem. Ref. Data.

[27] Yen G.C., Duh P.D., Tsai H.L. (2002). Antioxidant and pro-oxidant properties of ascorbic acid and gallic acid. Food Chem..

[28] Karadag A., Ozcelik B., Saner S. (2009). Review of methods to determine antioxidant capacities. Food Anal. Methods.

